# Hypertrophic Cardiomyopathy: The Time-Synchronized Relationship between Ischemia and Left Ventricular Dysfunction Assessed by Highly Sensitive Troponin I and NT-proBNP

**DOI:** 10.1155/2019/6487152

**Published:** 2019-06-20

**Authors:** Renata Rajtar-Salwa, Adam Gębka, Artur Dziewierz, Paweł Petkow Dimitrow

**Affiliations:** ^1^Second Department of Cardiology and Cardiovascular Interventions, University Hospital, Krakow, Poland; ^2^Second Department of Cardiology, Jagiellonian University Collegium Medicum, Krakow, Poland

## Abstract

The aim of this study was to compare NT-proBNP using the absolute values and NT-proBNP/ULN values that were standardized by age and gender between three subgroups: those without ischemia (negative hs-troponin I and no anginal pain (hsTnI-/AP-)), those with painless ischemia (hsTnI+/AP-), and those with painful ischemia (hsTnI+/AP+). Additionally, echocardiographic parameters were compared in these three subgroups. The absolute value of NT-proBNP was significantly higher in the painful ischemia subgroup (hsTnI-/AP- vs. hsTnI+/AP- vs. hsTnI+/AP+: 502 (174-833) vs. 969 (363-1346) vs. 2053 (323-3283) pg/ml; *p* = 0.018 for the whole-model analysis). The standardized value of NT-proBNP/ULN was gradually increased (hsTnI-/AP- vs. hsTnI+/AP- vs. hsTnI+/AP+: 3.61 + 0.63 vs. 6.90 + 1.31 vs. 9.35 + 1.87; *p* = 0.001 for the whole-model analysis). In the comparison between subgroups (hsTnI-/AP- vs. hsTnI+/AP- vs. hsTnI+/AP+), two echocardiographic parameters increased significantly. The left ventricular maximum wall thickness (LVMWT) at diastole was 1.99 ± 0.08 cm vs. 2.28 ± 0.13 cm vs. 2.49 ± 0.15 cm (*p* = 0.004 for the whole-model analysis). The maximal gradient of the provoked left ventricular outflow tract (LVOT) gradient increased significantly in only the painful-ischemia subgroup (11 (7-30) mmHg vs. 12 (9.35-31.5) mmHg vs. 100 (43-120) mmHg). In conclusion, both painless ischemia and painful ischemia are associated with a gradual, significant increase in NT-proBNP/ULN in comparison to the double-negative hsTnI/AP subgroup. In contrast, NT-proBNP is significantly higher in only the subgroup with painful ischemia.

## 1. Introduction

It is proposed [1, 2] that routine measurement biomarkers especially including [1] both N-terminal pro-B-type NT-pronatriuretic peptide (NT-proBNP) and cardiac troponin (Tn) may be useful in the clinical evaluation and management of patients with HCM. NT-proBNP is predominantly secreted from the ventricles in response to increased myocyte stretching from increased stress and pressure at the LV wall. It is plausible that microvascular ischemia directly stimulates the release of NT-proBNP in HCM. In clinical studies, there is little information about the combined use of Tn and plasma BNP as prognostic biomarkers for adverse events mediated by myocardial ischemia with LV dysfunction [3].

In a study by Kubo et al. [3] on 167 patients with HCM, TnI and BNP were measured only once at the initial examination without control measurements during a follow-up period of more than 3 years (mean value). Patients with elevated TnI values had more frequent adverse events. Similarly, the risk of adverse events was higher in patients with high BNP (≥200 pg/ml). Importantly, TnI used in combination with BNP further improved the prognostic value, as patients with high values of both cTnI and BNP had nearly 12 times higher risk of cardiovascular events than patients with a combination of low cTnI/BNP values. However, this study had some important limitations because there was a long time between the initial biomarker measurement and the final event.

In contrast, we studied the direct short-term relationship between angina pectoris and the levels of both hsTnI and NT-proBNP after a 24-hour monitoring period. Taking into account methodological aspects, we compared NT-proBNP using absolute values and NT-proBNP/ULN values that were standardized by age and gender between three subgroups: those without ischemia (negative hs-troponin I/no anginal pain (hsTnI-/AP-)), those with painless ischemia (hsTnI+/AP-), and those with painful ischemia (hsTnI+/AP+).

## 2. Methods

A total of 64 patients with HCM were recruited (mean age 37 ± 6 years, 33 men and 31 women). The study protocol was approved by the local institutional review board (Komisja Bioetyki Jagiellonian University KBET/119/B/2017). Informed consent was obtained from each participant. All patients met the standard diagnostic criteria for HCM [4]. In adults, HCM is defined by a wall thickness ≥ 15 mm in one or more LV myocardial segments that is not explained solely by loading conditions and measured by any imaging technique (echocardiography (our method), cardiac magnetic resonance imaging (CMR), or computed tomography (CT)) [4]. Patients on current pharmacotherapy or without pharmacotherapy (newly diagnosed patients referred to our ambulatory clinic) were examined by echocardiography with LVOT gradient provocation by a combination of two natural stimuli (orthostatic test and the Valsalva test).

The exclusion criteria were myocardial infarction with ST-segment or non-ST-segment elevation (current or previous), previous alcohol septal ablation, significant coronary stenosis in recent coronary angiography, diabetes mellitus, regular sports activity, dilated LV cavity and decreased LV contractibility (we included only patients with LVEF > 50%), atrial fibrillation, and elevated serum creatinine levels resulting in eGFR < 60 ml/min. We included only patients with coronary microvessel disease, which is a common abnormality in HCM at any age (inclusion criteria: normal/near-normal coronary arteries or no indication of coronary arteriography).

The minority of patients did not have coronary angiography performed (young, without AP, without risk factors for CAD—especially without diabetes mellitus—see below). The risk for CAD was minimal and coronary angiography was not indicated. Diabetes mellitus is usually linked with silent ischemia from epicardial coronary arteries (so it is necessary to exclude painless macrovascular stenosis).

Renal failure is a typical extracardiac factor related to TnI elevation. Frequent sports activity may be responsible for repetitive myocardial ischemia in some patients [5]. Patients were asked to report presence or absence of angina pectoris episodes before the 24-hour period. Next, an echocardiographic examination was performed. Just after, hsTnI and NT-proBNP (absolute or upper limit of normal (ULN)) were measured. There were 38 patients in subgroup 0 (i.e., the double-negative group; hsTnI-/AP-). Subgroup 1 (hsTnI+/AP-) was composed of 12 patients, and subgroup 2 (double positive) consisted of 14 patients. A cut-off value of 19 ng/l was used according to the manufacturer's instructions (bioMerieux VIDAS® High sensitive Troponin I). This value represents the 99th percentile of a presumably healthy population.

High-sensitivity troponin tests were performed with the use of the VIDAS High sensitive Troponin I (TNHS). The test is capable of measuring cardiac troponin I concentration in the range of 4.9-40,000.00 pg/ml (ng/1) without the need for dilution. The TNHS test was designed to meet the following criteria of repeatability and intralaboratory precision (Table 1).

The NT-proBNP tests were performed with use of an Elecsys proBNP II Cobas e601 system. The test is capable of measuring the NT-proBNP concentration in the range of 5–35,000 pg/ml without the need for dilution.

The proBNP II test was designed to meet the following criteria of repeatability and intralaboratory precision (Table 2).

The NT-proBNP levels were presented as absolute values and transformed values that were standardized according to sex and age based on the manufacturer's guidelines. (http://www.rochecanada.com/content/dam/roche_canada/en_CA/documents/package_inserts/ProBNPII-04842464190-EN-V9-CAN.pdf). Values NT-proBNP greater than the 95th percentile for age and gender (the ULN) were considered abnormal.

Therefore, the results were expressed as the ratio of the NT-proBNP to age and sex-matched ULN. Ratios > 1.0 were considered abnormal [6]. This standardization of NT-proBNP provides a normal distribution of data, whereas absolute values were distributed abnormally. In this situation, we do not need to perform a logarithmic transformation for artificial calculation.

For the statistical analysis, continuous variables were presented as the mean (±standard deviation (SD)) or median (interquartile range (IQR)). NT-proBNP levels were compared between the three subgroups of patients using the Kruskal–Wallis test, which was also used to compare the maximal LVOT gradient. The values of standardized NT-proBNP/ULN had a normal distribution according to the Kolmogorov-Smirnov test and were compared using ANOVA for comparison, which was also used to compare the LV maximum wall thickness (LVMWT) and left atrial diameter (LAD). *Stepwise multiple linear regression analysis was used to identify factors independently correlated with NT-proBNP levels. Patients' age, gender, troponin level, presence of angina, and several echocardiographic parameters (max LVH, resting LVOT gradient, maximal LVOT gradient, and LAD) were tested as possible candidates.* A *p* value of <0.05 was considered statistically significant (Statistica 12.0).

## 3. Results

Demographics, relevant echocardiographic information, medical history, and treatment data are presented in Table 3.

Among all patients, chest pain was present more than 10 hours (during daily physical activity on the first day) before the blood sampling at 8.00 a.m. on the second day in the morning. There were no chest pains during the night.

**Figure 1:**
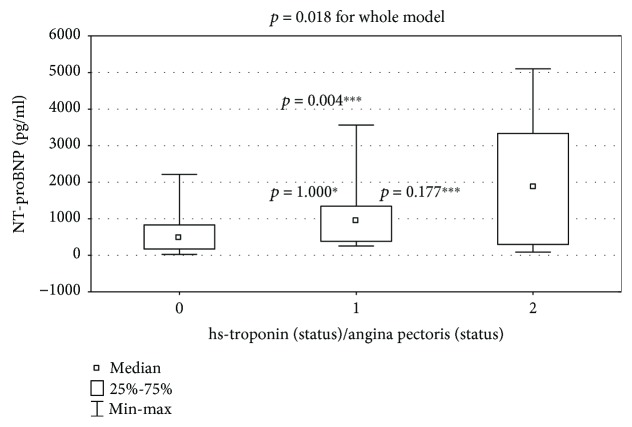
Comparison of NT-proBNP between subgroup 0 (hsTnI-/AP-), subgroup 1 (hsTnI+/AP-), and subgroup 2 (hsTnI+/AP+); *p* = 0.018 for the whole model. For inter-subgroup comparison: ^∗^subgroup 0 vs. 1: *p* = 1.000; ^∗∗^subgroup 1 vs. 2: *p* = 0.177; ^∗∗∗^subgroup 0 vs. 2: *p* = 0.004.

The absolute value of NT-proBNP was significantly higher in the painful ischemia subgroup (hsTnI-/AP- vs. hsTnI+/AP- vs. hsTnI+/AP+: 502 (174-833) vs. 969 (363-1346) vs. 2053 (323-3283) pg/ml; *p* = 0.0178 for the whole model, Figure 1).

**Figure 2:**
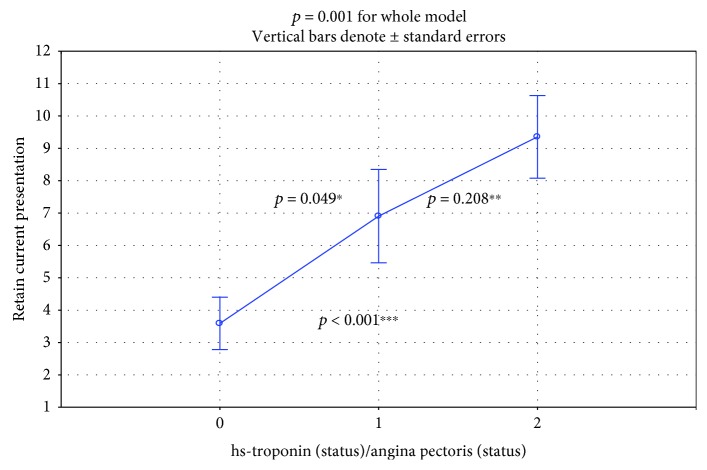
Comparison of NT-proBNP standardized according to age and sex between subgroup 0 (hsTnI-/AP-), subgroup 1 (hsTnI+/AP-), and subgroup 2 (hsTnI+/AP+). *p* = 0.001 for the whole model. Inter-subgroup comparison: ^∗^subgroup 0 vs. 1: *p* = 0.049; ^∗∗^subgroup 1 vs. 2: *p* = 0.208; ^∗∗∗^subgroup 0 vs. 2: *p* < 0.001.

The standardized value of NT-proBNP/ULN showed a gradual significant increase (hsTnI-/AP- vs. hsTnI+/AP- vs. hsTnI+/AP+: 3.59 + 0.63 vs. 6.90 + 1.31 vs. 9.35 + 1.87; *p* = 0.001 for the whole model in ANOVA; Figure 2).

**Figure 3:**
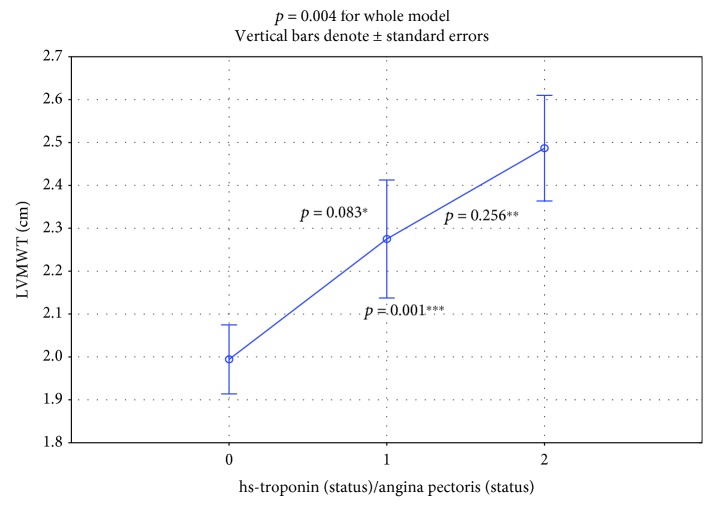
Significant increase (*p* = 0.004) of LVMWT in the whole model. Inter-subgroup comparison: ^∗^subgroup 0 vs.1: *p* = 0.083; ^∗∗^subgroup 1 vs. 2: *p* = 0.256; ^∗∗∗^subgroup 0 vs. 2: *p* = 0.001.

In the comparison between subgroups (hsTnI-/AP- vs. hsTnI+/AP- vs. hsTnI+/AP+), two echocardiographic parameters increased significantly. The LV maximum wall thickness (LVMWT) at diastole was 1.99 ± 0.08 cm vs. 2.28 ± 0.13 cm vs. 2.49 ± 0.15 cm (Figure 3, normal distribution, ANOVA test, *p* = 0.004 in the whole-model analysis).

**Figure 4:**
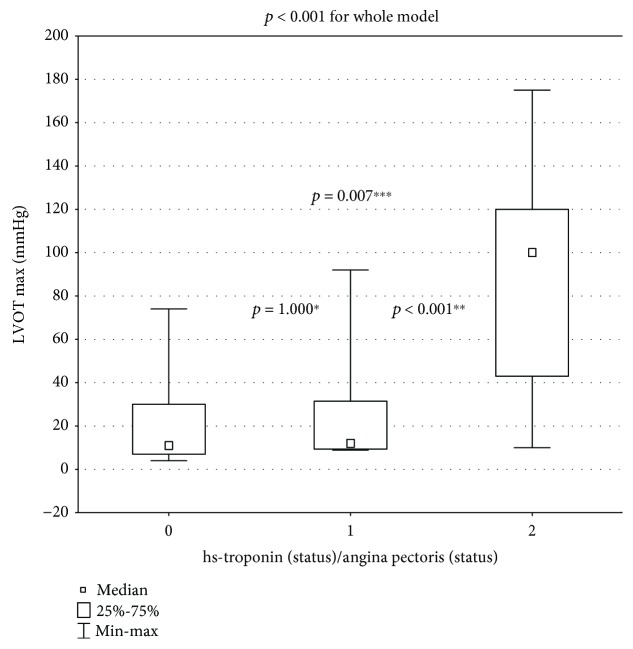
Significant increase (*p* < 0.001 for the whole model) of provocable LVOT gradient. Inter-subgroup comparison: ^∗^subgroup 0 vs. 1: *p* = 1.000; ^∗∗^subgroup 1 vs. 2: *p* < 0.001; ^∗∗∗^subgroup 0 vs. 2: *p* = 0.007.

The maximal provoked LVOT gradient increased significantly in only the painful-ischemia subgroup: 11 (7-30) mmHg vs. 12 (9.35-31.5) mmHg vs. 100 (43-120) mmHg (Figure 4; abnormal distribution: Kruskal–Wallis test, *p* < 0.001 for the whole model).

**Figure 5:**
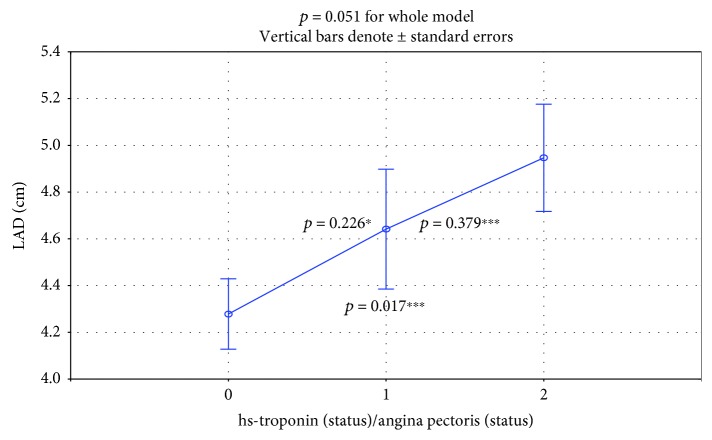
There was a nonsignificant increase in LAD (*p* = 0.051 for the whole model). ^∗^Subgroup 0 vs. 1: *p* = 0.226; ^∗∗^subgroup 1 vs. 2: *p* = 0.379; ^∗∗∗^subgroup 0 vs. 2: *p* = 0.017.

The increase of LAD from subgroup to subgroup was nonstatistically significant (4.28 ± 0.16 vs. 4.64 ± 0.28 vs. 4.95 ± 0.15; *p* = 0.051 for the whole model, Figure 5).

NT-proBNP/ULN was more strongly correlated with echocardiographic parameters than NT-proBNP (Table 4).

In multiple linear regression analysis, resting LVOT gradient, LAD, and the presence of angina were identified as independent factors affecting NT-proBNP levels in patients with HCM (Table 5).

## 4. Discussion

hsTnI is a precise and very useful biomarker for the detection of even small, focal, subendocardial myocardial injury caused by ischemia in patients with HCM. In recent investigations, measurements of hsTnI levels were synchronized with a noninvasive assessment of clinical and hemodynamic parameters within a short time, similar with previous studies [7–11]. We have documented that levels of both NT-proBNP and NT-proBNP/ULN were the highest in the most ischemic subgroup (painful angina pectoris; hsTnI+/AP+). Both biomarkers and echocardiographic parameters have not been investigated previously using currently proposed models of analysis [12–15].

In the hsTnI+/AP+ subgroup, both LVMWT at diastole and the provocable LVOT gradient had significantly higher values. In the subgroup of painless ischemia, the LVMWT at diastole had an intermediate value and differed significantly from the values of the nonischemic and painful ischemic subgroups (Figure 3). Our findings are rational because both increased myocardial mass and LVOT gradient induce myocardial ischemia through an increase in oxygen demand. LAD is the third echocardiographic risk factor for sudden death included in the guideline calculator from the European Society of Cardiology [4, 10, 11]. This value was increased in subgroups, but the differences were only on the statistical borderline (*p* = 0.052, Figure 5).

In an experimental protocol for HCM with more physiological conditions [15], biomarkers were measured before exercise testing with only one control point at 4 hours postexercise. NT-proBNP increased by 27% after exercise. Similarly, hsTnI increased by 24% 4 hours after exercise, but the differences were not statistically significant. The 4-hour check-point in the postexercise recovery seems to be too long for peak NT-proBNP and too short for peak hsTnI values. In small study with 7 young HCM patients without symptomatic coronary artery disease, authors [16] detected elevated troponin levels after physical exercise in 5 patients. In serial measurement, the peak concentration had been reached between 6 and 9 hours and levels returned to preexercise values within 24 hours. Troponin release was consistently diminished after use of a beta-blocker. We tried to study this problem by using a 24-hour spectrum of time and with a more physiological approach. We monitored the 24-hour physical activity and occurrence of angina. It has been proposed that moderate, fluctuating exercise during daily physical activity may be more appropriate to detect abnormalities in cardiac biomarker release, rather than maximum symptom-limited exercise [15]. To support the theory about the link with postexercise prolonged myocardial ischemia, we need a larger study, with many points in time to measure hsTnI (24-hour profile of release). Tesic et al. [17] recently found that the coronary flow reserve in the left anterior descending artery appeared to be an independent predictor of NT-proBNP. Thus, elevated NT-proBNP might be the result of cardiac ischemia indicated by low coronary flow reserve.

### 4.1. Limitations

The main limitation of study is the relatively small number of patients due to several exclusion criteria. Not all of the patients underwent coronary arteriograms, which was only performed on patients with the appropriate indications. The minority of patients did not have coronary angiography performed (young, without AP, and without risk factors for CAD). The risk for CAD was minimal and coronary angiography was not indicated. The strategy of noninvasive identification of subgroups with low likelihood of obstructive CAD [18] is effective.

The next limitation of the study was the following problem. We measured biomarkers simultaneously, once in time, and we do not have a sufficient period of follow-up with prognostic findings.

## 5. Conclusions

Both painless ischemia and painful ischemia are associated with a gradual, significant increase in NT-proBNP/ULN in comparison to the double-negative hsTnI/AP subgroup. In contrast, NT-proBNP was significantly higher in only the subgroup with painful ischemia. In the comparison between subgroups (hsTnI-/AP- vs. hsTnI+/AP- vs. hsTnI+/AP+), two echocardiographic parameters increased significantly: LVMWT at diastole (in the whole-model analysis) and the maximal provoked LVO T gradient (only in the painful subgroup in both analysis models).

## Figures and Tables

**Table 1:** TNHS test repeatability and intralaboratory precision.

High-sensitivity troponin (pg/ml; ng/l)	Assay output requirements	
	Repeatability (within the same series) (CV (%))	Intralaboratory precision (total) (CV (%))
9-20	≤10%	≤12%
>20	≤10%	≤10%

Precision was assessed in accordance with the EP05-A3 protocol of the Clinical and Laboratory Standards Institute (CLSI): “Evaluation of Precision of Quantitative Measurement Procedures; Approved Guidelines—Third Edition” (CLSI Document EP05-A3).

**Table 2:** NT-proBNP test repeatability and intralaboratory precision.

NT-proBNP		Assay output requirements	
pg/ml	pmol/l	Repeatability (within the same series) (CV (%))	Intralaboratory precision (total) (CV (%))
100-500	11.8–59.0	≤5%	≤8%
>500	>59,0	≤7%	≤10%

Precision was assessed in accordance with the EP05-A3 protocol of the Clinical and Laboratory Standards Institute (CLSI): “Evaluation of Precision of Quantitative Measurement Procedures; Approved Guidelines—Third Edition” (CLSI Document EP05-A3).

**(a):** 

Mean age	37 ± 6 years
Males/females	33/31
The ICD implantation	8 patients
EF %	59 ± 8%
hsTnI value	73.74 ± 232.9
Medications	Patients
Beta-blockers	43
Verapamil	17
Diuretics	3
ACE inhibitors	4
Past history of AP in years	4.1 + 1.2 years

**(b):** 

		Total (*N* = 64)	Subgroup 0 (*N* = 38)	Subgroup 1 (*N* = 12)	Subgroup 2 (*N* = 14)
Baseline characteristics of subgroups of patients with HCM					
NYHA	Class I (*n* (%))	12 (19%)	9 (24%)	3 (25%)	0^∗^^,^^∗∗^
	Class II (*n* (%))	33 (51%)	20 (52%)	6 (50%)	7 (50%)
	Class III (*n* (%))	19 (30%)	9 (24%)	3 (25%)	7 (50%)∗
CCS	Class I (*n* (%))	24 (38%)	18 (47%)	5 (42%)	1 (7%)^∗^^,^^∗∗^
	Class II (*n* (%))	29 (45%)	16 (42%)	5 (42%)	8 (57%)
	Class III (*n* (%))	11 (17%)	4 (11%)	2 (8%)	5 (36%)^∗^
Syncope (*n* (%))		25 (39%)	13 (34%)	5 (42%)	7 (50%)
Sudden death in family history (*n* (%))		23 (36%)	14 (37%)	4 (33%)	5 (36%)
All patients had Holter					
NSVT in Holter (*n* (%))		26 (41%)	15 (39%)	5 (42%)	6 (43%)
LV maximal wall thickness (LVMWT) at diastole (cm)		2.23 ± 0.57	Detailed calculation in Figure 3		
Resting LVOT gradient, ≥30 mmHg (*n* (%))		14 (22%)	6 (16%)	3 (25%)	5 (36%)
Provocable LVOT gradient, ≥30 mmHg (*n* (%))		14 (22%)	5 (13%)	3 (25%)	6 (43%)^∗^
Left atrial diameter (cm)		4.78 ± 0.64	Detailed calculation in Figure 5		

Abbreviations: CCS: Canadian Cardiovascular Society; LVOT: left ventricular outflow tract; LV: left ventricular; NSVT: nonsustained ventricular tachycardia; NYHA: New York Heart Association. ^∗^*p* < 0.05 subgroup 0 vs. 2; ^∗∗^*p* < 0.05 subgroup 1 vs. 2.

**Table 4:** Correlations between NT-proBNP(/ULN) and echocardiographic parameters.

	NT-proBNP	NT-proBNP/ULN
LVMWT	*r* = 0.24^∗^	*r* = 0.36
LVOTG max	*r* = 0.44	*r* = 0.54
LAD	*r* = 0.41	*r* = 0.42

^∗^Nonsignificant; remaining correlations were significant; *p* < 0.05.

**Table 5:** Results of linear regression analysis.

Independent variable	Coefficient	Standard error	Standardized coefficient	*p* value
Resting LVOT gradient	19.76	5.29	0.41	<0.001
LAD	314.44	123.30	0.26	0.013
Angina	658.35	297.13	0.25	0.031

## Data Availability

The data used to support the findings of this study are available from the corresponding author upon request.

## Additional Points

*Clinical Perspective*. The study documented the evidence that positive troponin status is timely related with higher biomarker value NT-proBNP reflecting LV myocardial dysfunction. From the clinical perspective, monitoring of these 2 biomarkers may improve pharmacological and nonpharmacological treatments and safety of exercise training.
